# Germline genetic variations in *PDZD2* and *ITPR2* genes are associated with clear cell renal cell carcinoma in Chinese population

**DOI:** 10.18632/oncotarget.6917

**Published:** 2016-01-14

**Authors:** Ning Zhang, Yishuo Wu, Jian Gong, Kaiwen Li, Xiaolin Lin, Haitao Chen, Yang Yu, Yuancheng Gou, Jiangang Hou, Deke Jiang, Rong Na, Xiang Wang, Qiang Ding, Jianfeng Xu

**Affiliations:** ^1^ Department of Urology, Huashan Hospital, Fudan University, Shanghai, PR China; ^2^ Fudan Institute of Urology, Huashan Hospital, Fudan University, Shanghai, PR China; ^3^ Department of Urology, Sun Yat-sen Memorial Hospital, Sun Yet-sen University, Guandong, PR China; ^4^ State Key Laboratory of Genetic Engineering, School of Life Sciences, Fudan University, Shanghai, PR China; ^5^ Center for Genetic Epidemiology, School of Life Sciences, Fudan University, Shanghai, PR China; ^6^ Health Communication Institute, School of Public Health, Fudan University, Shanghai, PR China; ^7^ Program for Personalized Cancer Care, NorthShore University HealthSystem, Evanston, IL, USA

**Keywords:** renal cell carcinoma, SNPs, genome-wide association, Chinese

## Abstract

Genome-wide association studies (GWAS) of renal cell carcinoma (RCC) have identified single nucleotide polymorphisms (SNPs) associated with RCC in European and African American population. In this study, we evaluated whether these SNPs are associated with clear cell RCC (ccRCC) in Chinese population. All reported RCC risk-associated SNPs from GWAS were evaluated in 346 ccRCC cases and 1,130 controls. Rs10054504 (at *PDZD2*, Odds ratio, OR = 0.71, 95%CI:0.59-0.86, *P* = 0.0006), rs718314 (at *ITPR2*, OR = 0.56, 95%CI:0.45-0.69, *P* = 5.26 × 10^−8^) and rs1049380 (at *ITPR2*, by dominant model, OR = 1.58, 95%CI:1.18-2.13, *P* = 0.0025) were significantly associated with ccRCC risk in Chinese population. To conclude, genetic variations in *PDZD2* and *ITPR2* are ccRCC-risk associated in Chinese population.

## BACKGROUND

With an approximately 270,000 new cases and 116,000 deaths every year worldwide, kidney cancer has become one of the most common malignancies and accounts for about 2% of all cancers [1]. In 2012, the estimated new cases of kidney cancer were 213,900 for male only, which indicated the rapid increase of the incidence [2]. Renal cell carcinoma (RCC) represents nearly 90% of all kidney cancers. For adult kidney cancer, clear cell RCC (ccRCC) is the most common type.

The risk factors of RCC have been confirmed including smoking, obesity and hypertension; however, it is believed that the factors were interacted together with genetic predisposition to cause the disease. In addition, a fist-degree relative with RCC is also considered as an important risk factor, which indicated the inherited risk of RCC [3].

Several Genome-wide association studies (GWAS) were performed to evaluate the genetic variants in patients with RCC. Over 10 single nucleotide polymorphisms (SNPs) have been observed significantly or potentially associated with RCC [4–8]. They may have cumulative effect on RCC risk.

The main treatment of RCC is resection by surgical procedure including radical nephrectomy or partial nephrectomy. Poorer prognosis usually occurs in high stage non-resectable diseases. Therefore, early diagnosis for high risk individual is important. Testing the germline mutation of RCC risk SNPs is one of the useful methods to identify people with high RCC risks in addition to environmental risk factors.

However, in China, there is limited information regarding RCC risk SNPs. Therefore, the objective of this study is to evaluate the 12 RCC risk-associated SNPs identified from GWAS in a case-control study (ccRCC vs. control) based on Chinese population.

## RESULTS

A total of 346 ccRCC cases were finally included in this study, of which 230 (66.5%) were male and 114 (32.9%) were female. Table 1 listed the baseline characteristics of the patients. The mean age of the cases is 55.85±12.45. The mean tumor size of ccRCC was 3.63±2.42. One hundred and fifty-five patients were diagnosed having left ccRCC, while 158 had right ccRCC. Two hundred and two (58.4%), 69 (19.9%), 22 (6.4%), 0 (0%) and 53 (15.3%) had T1, T2, T3, T4 and Tx tumor respectively. Two cases were observed having metastasis (M+), and two were observed having positive lymph nodes (N+). Eleven (3.2%), 120 (34.7%), 40 (11.6%) and 1 (0.3%) patients had Fuhrman Grade 1, 2, 3 and 4 ccRCC respectively. Missing phenotype of Fuhrman Grade was found in 174 patients.

**Table 1:** Characteristics of study subjects

Variables	Case	Control	P-value
N of cases	346	1130	-
Age (Mean±SD)^a^	55.85±12.45	51.20±9.70	0.0001
Gender, # (%)			0.52
Male	230 (66.5)	732 (64.8)	-
Female	114 (32.9)	398 (35.2)	-
Location of Tumor, # (%)			
Left Kidney	155(46.5)	-	-
Right Kidney	158 (47.4)	-	-
Missing	20 (6.0)	-	-
Tumor Size (Mean±SD)	3.63±2.42	-	-
T-stage, # (%)			
T1	202 (58.4)	-	-
T2	69 (19.9)	-	-
T3	22 (6.4)	-	-
T4	0	-	-
Tx or Missing	53 (15.3)	-	-
M-stage, # (%)			
M0	325 (93.9)	-	-
M1	2 (0.6)	-	-
Mx or Missing	19 (5.5)	-	-
N-stage, # (%)			
N0	325 (93.9)	-	-
N1	2 (0.6)	-	-
Nx or Missing	19 (5.5)	-	-
Fuhrman Grade, # (%)			
1	11 (3.2)	-	-
2	120 (34.7)	-	-
3	40 (11.6)	-	-
4	1 (0.3)	-	-
Missing	174 (50.3)	-	-

a: Age at diagnosis for cases or at recruitment for controls.

All the SNPs of control group passed Hardy-Weinberg equilibrium test. All the SNPs are polymorphic in Chinese population. For the 12 SNPs, rs10054504 (Odds ratio, OR = 0.71, 95%CI:0.59-0.86, *P* = 0.0006) and rs718314 (OR = 0.56, 95%CI:0.45-0.69, *P* = 5.26×10^−8^) were significantly associated with ccRCC risk in Chinese population (Table 2). Rs1049380 (*P* = 0.020) did not reach Bonferroni correction significant level using additive model; however, it was significant associated with ccRCC (OR = 1.58, 95%CI:1.18-2.13, *P* = 0.0025) when being analyzed by dominant model (Table 2).

**Table 2:** Results of association test in Chinese population for reported RCC risk-associated SNPs

Origin of GWAS	Chr	SNP	Gene	Position	Minor/Major Alleles	ccRCC vs. Controls							
						F_A	F_U	OR	Lower 95%CI	Upper 95%CI	P-value(A)^a^	P-value(D) ^a^	P-value(R) ^a^
European	2	rs11894252	EPAS1	46533376	C/T	0.144	0.113	1.36	1.05	1.77	0.02	0.04	0.06
European	2	rs1867785	EPAS1	46534338	G/A	0.142	0.123	1.19	0.92	1.54	0.18	-	-
European	2	rs7579899	EPAS1	46537604	G/A	0.146	0.145	1.01	0.79	1.30	0.91	-	-
European	2	rs12105918	ZEB2	145208193	C/T	0.096	0.083	1.16	0.87	1.55	0.32	-	-
European	2	rs13389578	ZEB2	145216048	C/T	0.083	0.077	1.09	0.81	1.48	0.57	-	-
European	5	rs10054504	PDZD2	32000483	C/T	0.295	0.365	0.71	0.59	0.86	0.0006	-	-
European	8	rs35252396	PVT1	128889372	AC/CG	0.454	NA^b^	-	-	-	-	-	-
Europan/African American	11	rs7105934	-	69239741	A/G	0.047	0.067	0.68	0.46	1.01	0.06^c^	-	-
European	12	rs718314	ITPR2	26453283	A/G	0.192	0.303	0.56	0.45	0.69	5.26×10^−8^	-	-
European	12	rs1049380	ITPR2	26489544	A/C	0.519	0.469	1.58^d^	1.18^d^	2.13^d^	0.020	0.0025	0.49
African American	12	rs10771279	ITPR2	26530543	T/C	0.296	0.303	0.96	0.80	1.17	0.71	-	-
European	12	rs4765623	SCARB1	125320850	T/C	0.434	0.413	1.09	0.92	1.29	0.33	-	-

F_A: Frequency of affected (Case); F_U: Frequency of unaffected (Control); OR: Odds ratio; CI: Confidence interval

a: A stands for additive model; D stands for dominant mordel; R stands for recessive model.

b: The frequency of this locus was not able to be genotyped or imputed in control population.

c: The P value was calculated by Fisher exact test.

d: The OR and 95%CI of OR were calculated using dominant model.

We also evaluated the association between SNPs and tumor size, SNPs and T staging, SNPs and Fuhrman grade. None of the SNPs were significantly associated with tumor size and Fuhrman grade (all *P* > 0.05, Supplementary Table 2). Rs10771279 were found associated with T staging of ccRCC (*P* = 0.047, Beta = 0.11, SE = 0.014, Supplementary Table 2), however, did not reach the Bonferroni correction significant *P* value of 0.0042.

## DISCUSSION

Family history can provide genetic risk information of the disease, however, is uninformative in China. This is because: (1) the family history of cancers was extremely low in China because of the poor healthcare policy that limited the detection of diseases in past decades; (2) the incomplete cancer registration policy in the past; (3) the family planning policies may interfere the existence of affected first-degree relative. Therefore, it is particularly important to perform risk assessment of ccRCC by using inherited genetic markers in China. In the current study, we provided the genetic risk information in Chinese population by evaluating the risk SNPs from GWAS of RCC based on other races. Three SNPs were significantly associated with ccRCC.

Rs10054504 (5p13.3) was first identified in European population having potential association with RCC [7]. It is located in the intron 4 of *PDZD2* (PDZ domain containing 2). Proteins containing this PDZ domain have tumor suppressive actions by transcriptional regulating p53 activation when secreted [9]. In the current study, carrying T allele on this locus might increase the risk of having ccRCC (OR = 1/0.71 = 1.41). We hypothesized that the C allele of rs10054504 on the intron 4 might decrease the expression of *PDZD2*, which suppressed the activation of p53 and inhibited the tumor suppressive actions.

Rs1049380 is located in the 3′-untranslated region of *ITPR2* (12p11.23), while rs718314 was about 36kb downstream of rs1049380 in the intergenic region. These two SNPs have strong linkage disequilibrium (LD, R^2^ = 0.58, Supplementary Figure 1). A previous study showed that ITPR2 protein was increased in kidney tissue of mice when using nephrotoxic agent [10]. Another study suggested that in RCC, *ITPR2* expression was decreased [11]. In this study, risk allele A could increase the risk of ccRCC. The previous GWAS study showed the same result that major allele in European population (which is A allele according to HapMap-CEU data) might increase the risk of ccRCC (OR = 1.18, 95%CI: 1.12-1.25) [5]. Therefore, we hypothesized that with the risk allele A at 3′-untranslated region, expression of *ITPR2* might be inhibited, causing the carcinogenesis of ccRCC. However, the most recent study indicated that C allele of rs1049380 was associated with early onset of RCC [12]. Nevertheless, these results indicated the strong association between rs1049380 and ccRCC. Further functional studies should perform to validate this hypothesis.

Rs35252396 failed to be genotyped or imputed in control group. Probably because that rs35252396 (AC/CG, Chr 8) is a cluster of two SNPs. The frequency information is not available in 1000 Genomes Project database. However, SNP coded as rs6470588 (A/C) at the same position represented the first allele of rs35252396. We used the frequency information (A allele frequency is 0.402) of rs6470588 (HapMap-CHB) as control to estimate the association between rs35252396 and ccRCC (strong LD between two alleles). However, no significant association (*P* = 0.71) was observed.

Several limitations of the current study should be noted. 1) The association between rs10771279 and T staging of the tumor (*P* = 0.047) did not attain statistical significance based on Bonferroni correction (corrected significant *P* = 0.0042). However, Bonferroni correction might be too stringent since not all of tested SNPs were independent. Tested SNPs had high LD, for example, rs718314 and rs1049380 had a LD of R^2^ = 0.58. Because of the lack of T4 and M+ samples in the current study, the association between SNPs and disease staging should be further evaluated. 2) The mean age of control group was about 4 years younger than RCC group (*P* < 0.001). After adjusting for age by logistic regression, these three RCC risk SNPs (rs10054504, rs718314 and rs1049380) remained significant (Supplementary Table 1). 3) We realized that different method of genotyping and the imputation could bring biases. [13] To address this problem, we randomly chose 360 controls to performed quality control by using the same methods of genotyping as using in case group. We genotyped these control samples by using MassARRAY iPLEX system for SNPs other than rs13389578, rs1049380 and rs10054504. For SNPs rs13389578, rs1049380 and rs10054504, TaqMan MGB technology was used. Four samples were failed to genotyped (call rate 98.88%). We then compared the concordance rate between methods (alleles failed to be called were excluded when calculating, Supplementary Table 3). Briefly, the concordance rates among different genotyping platforms and imputation were >99.79%. We thus believe that the differences among platforms were limited. 4) This study was based on a population from a single institute. However, as a tertiary medical center in Shanghai, China. Patients around the country seek their service for its high quality of healthcare. 5) The current study could only detect modest effects for binary traits because of the relatively small sample size. For example, there was 80% power to detect an OR of 1.45-1.30, assuming a mean minor allele frequency of 0.1-0.5 for case-control analysis. However, the power was sufficient to detect relatively small magnitudes of the association between SNPs and diseases. Thus, SNPs that were not significantly associated with RCC in the current study were not the genetic risk in Chinese population. Further replication studies are needed to confirm our results.

## CONCLUSIONS

In conclusion, we systematically evaluating RCC risk-associated SNPs indentified from previous GWAS in a Chinese population, finding that one SNP located in *PDZD2* and two SNPs located in *ITPR2* were ccRCC-risk associated in Chinese population. The result might provide evidence for identifying high risk individual and improving early detection of ccRCC.

## MATERIALS AND METHODS

### Study population and study design

All patients with ccRCC (cases) were recruited from 2010 to 2014 in our institute (Huashan Hospital, Fudan University, Shanghai, China). The inclusion criteria were: (1) Patients with surgical resectable kidney tumor (received radical nephrectomy or partial nephrectomy via open procedure or laparoscopic procedure because of kidney tumor); (2) the specimens were diagnosed as ccRCC by Department of Pathology in our institute (all the specimens were reviewed by the same group of pathologists.); (3) Blood samples were collected. Clinical information was collected. Patients who were diagnosed as other types of kidney tumor, had missing blood samples or missing pathological diagnosis (whether the tumor is RCC or not) were excluded. A total of 1,130 people were included in the control group from community populations. The characteristics of the population from control group were reported in previous study.[14] Written informed consent was obtained from each patient. The study was approved by institutional review board of Huashan Hospital, Fudan University, Shanghai, China.

### SNP selection and genotyping

All established or potential RCC risk-associated SNPs from published GWAS of RCC in European and African American descent were selected for this study. Among 12 SNPs, 11 SNPs [4, 5, 7] and 2 SPNs [6, 8] were significantly or potentially associated with ccRCC in European population and African American population, respectively. The characteristics of SNPs that were associated with ccRCC in previous GWAS were showed in Table 3.

**Table 3:** Reported RCC risk-associated SNPs from GWAS studies of European and African American population

Chr	References	Origin of GWAS	SNP	Gene	Position	OR (95% CI)	P value
2	Mark P. Purdue et al.^4^	European	rs11894252	EPAS1	46533376	1.14 (1.09-1.20)	1.8×10^−8^
2	Mark P. Purdue et al.^4^	European	rs1867785	EPAS1	46534338	LD with rs11894252	
2	Mark P. Purdue et al.^4^	European	rs7579899	EPAS1	46537604	1.15 (1.10-1.21)	2.3×10^−9^
2	Marc Henrion et al.^7^	European	rs12105918	ZEB2	145208193	-	1.8×10^−8^
2	Marc Henrion et al.^7^	European	rs13389578	ZEB2	145216048	-	2.14×10^−7^
5	Marc Henrion et al.^7^	European	rs10054504	PDZD2	32000483	-	7.68×10^−7^
8	Julius Gudmundsson et al.^6^	European	rs35252396	PVT1	128889372	1.27 (1.18-1.37)	5.4×10^−11^
11	Mark P. Purdue et al.^4′8^	Europan/African American	rs7105934	-	69239741	0.69 (0.62-0.76)	7.8×10^−14^
12	Wu Xifeng et al.^5^	European	rs718314	ITPR2	26453283	1.19 (1.13-1.26)	8.89×10^−10^
12	Wu Xifeng et al.^5^	European	rs1049380	ITPR2	26489544	1.18 (1.12-1.25)	6.07×10^−9^
12	Mark P. Purdue et al.^8^	African American	rs10771279	ITPR2	26530543	0.48 (0.36-0.63)	1.2×10^−7^
12	Mark P. Purdue et al.^4^	European	rs4765623	SCARB1	125320850	1.15 (1.09-1.20)	2.6×10^−8^

RCC: Renal cell carcinoma; GWAS: Genome-Wide Association Studies.

Whole-genome DNA was isolated and purified from leucocytes of blood samples from each patient. DNA was extracted by using Puregene DNA Purification Kit (case) and Qiagen QIAamp DNA Blood Mini Kit (control). SNPs were genotyped by using MassARRAY iPLEX system (Sequenom, Inc, San Diego, CA) at Fudan University, Shanghai, China. Duplicates from four subjects and four negative controls (water samples) were included in the 384-well plate for genotyping quality control. Three SNPs (rs13389578, rs1049380 and rs10054504) which were not able to genotyped by the Sequenom system (because of the conflict of promoters among SNPs) were genotyped by TaqMan MGB technology (Applied Biosystems, Foster City, CA). All the control samples were previously performed GWAS by using Illumina Human OmniExpress Bead Chips. SNPs that were not included in the GWAS chip (rs11894252, rs7579899, rs7105934 and rs4765623) were imputed based on haplotype data from the 1000 Genomes Project CHB+JPT subjects (Phase I integrated data version 3, released Mar 2012) by using IMPUTE2.2.2 program. A posterior probability of >0.90 was applied to call imputed genotypes. Genotype data were not available for rs35252396 in control group which was not included in the GWAS chip and was not able to impute. All assays were performed in blinded fashion.

### Statistic analysis

Each SNP was tested for Hardy-Weinberg equilibrium. The association between SNPs and ccRCC were evaluated by using logistic regression model or Fisher's exact test if the allele frequency of SNP was lower than 5%. Additive model of inheritance was used for all of these analyses. For SNPs that were significant at a liberal criterion of *P* = 0.05, dominant and recessive models were also tested. The associations between SNPs and tumor size, SNPs and T staging, SNPs and Fuhrman grade were evaluated by using Fisher's exact test. The analysis was performed using PLINK 1.09 with 2-tailed P values. The Bonferroni correction P value of 0.0042 (0.05/12) was considered statistically significant.

## SUPPLEMENTARY MATERIALS FIGURE AND TABLES


