# Supplementary material for: Germline genetic variations in PDZD2 and ITPR2 genes are associated with clear cell renal cell carcinoma in Chinese population
Source: Oncotarget. 2016 Jan 14;8(15):24196–201. doi: 10.18632/oncotarget.6917 (PMC5421839; doi:10.18632/oncotarget.6917)
Supplement: Supplementary file 1 [file oncotarget-08-24196-s001.pdf]

Germline genetic variations in *PDZD2* and *ITPR2* genes were associated with clear cell renal cell carcinoma in chinese population

Supplementary Material

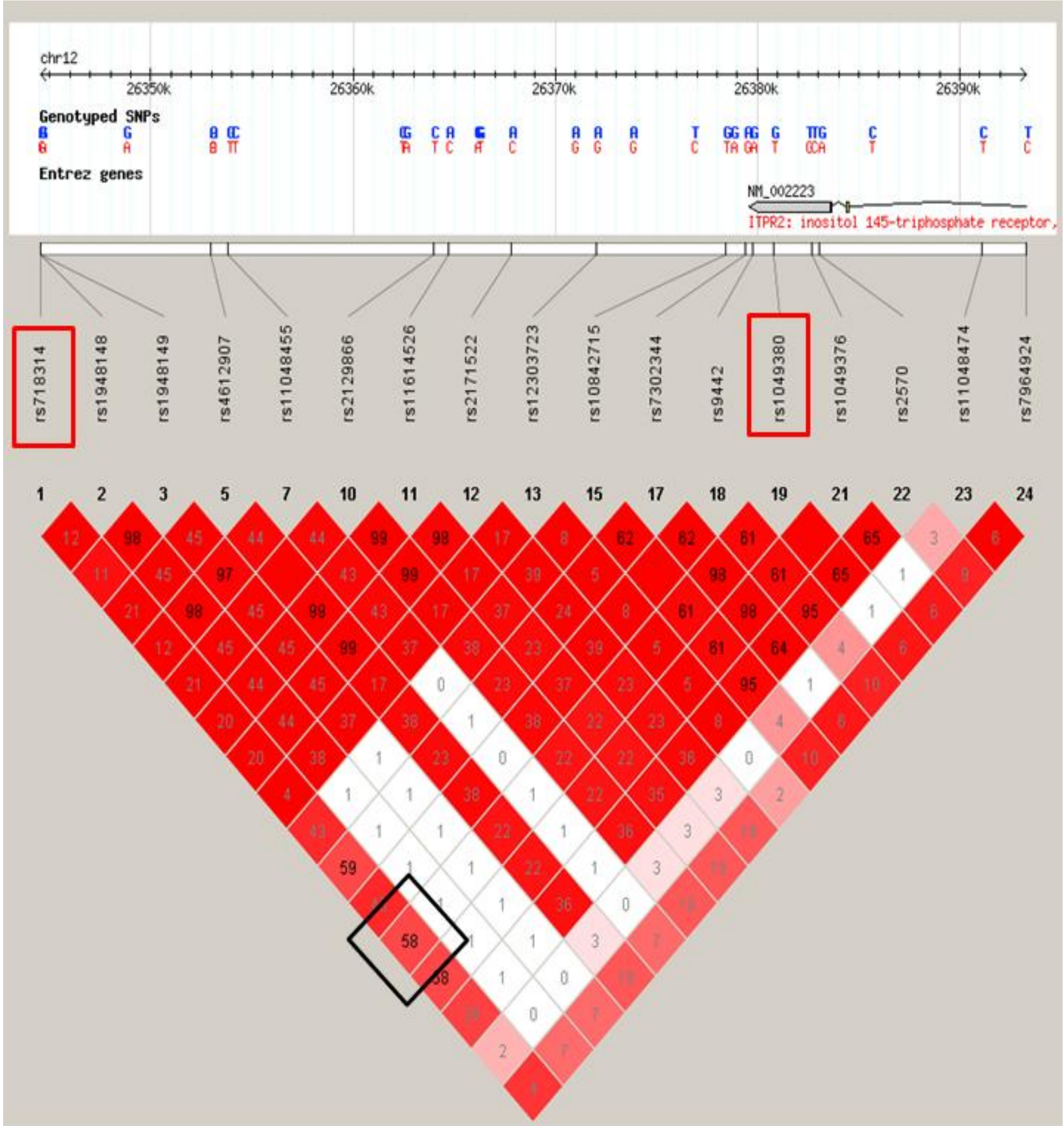

Supplementary Figure 1. Linkage Disequilibrium (LD) between rs718314 and rs1049380 (R^2=0.58) by using Hapmap 3 Rel 27 (CHB) data. Red color stands for D' value. Number stands for R^2 value.

Supplementary Table 1. Results of association test in Chinese population for reported RCC risk-associated SNPs adjusting different valuables.

Origin of GWAS	Chr	SNP	Gene	Position	Minor/Major Alleles	ccRCC vs. Controls Adjusted by age	
						OR	P-value
European	2	rs11894252	*EPAS1*	46533376	C/T	1.35	0.03
European	2	rs1867785	*EPAS1*	46534338	G/A	1.17	0.23
European	2	rs7579899	*EPAS1*	46537604	G/A	1.01	0.93
European	2	rs12105918	*ZEB2*	145208193	C/T	1.72	0.30
European	2	rs13389578	*ZEB2*	145216048	C/T	1.09	0.60
European	5	rs10054504	*PDZD2*	32000483	C/T	0.71	**0.0008**
European	8	rs35252396	*PVT1*	128889372	AC/CG	NA[b]	-
Europan/African American	11	rs7105934	-	69239741	A/G	0.70	0.08
European	12	rs718314	*ITPR2*	26453283	A/G	0.55	**4.48×10^{-8}**
European	12	rs1049380	*ITPR2*	26489544	A/C	1.62[b]	**0.002** [b]
African American	12	rs10771279	*ITPR2*	26530543	T/C	0.94	0.53
European	12	rs4765623	*SCARB1*	125320850	T/C	1.08	0.41

OR: Odds ratio

a: The frequency of this locus was not able to be genotyped or imputed in control population.

b: The association test of this SNP was evaluated by dominant model.

Supplementary Table 2. Results of association tests in Chinese population between SNPs and tumor size, T staging and Fuhrman grade.

SNP	Gene	Position	Minor/Major Alleles	Tumor Size		T Staging		Fuhrman grade	
				Beta[a]	P[a]	Beta[a]	P[a]	Beta[a]	P[a]
rs11894252	*EPAS1*	46533376	C/T	0.24	0.36	0.13	0.09	0.10	0.20
rs1867785	*EPAS1*	46534338	G/A	0.27	0.31	0.13	0.08	0.07	0.42
rs7579899	*EPAS1*	46537604	G/A	0.24	0.37	0.12	0.12	0.10	0.21
rs12105918	*ZEB2*	145208193	C/T	0.005	0.99	-0.04	0.68	-0.006	0.95
rs13389578	*ZEB2*	145216048	C/T	0.11	0.74	-0.004	0.96	-0.08	0.49
rs10054504	*PDZD2*	32000483	C/A	-0.36	0.17	-0.13	0.09	-0.03	0.74
rs35252396	*PVT1*	128889372	AC/CG	0.29	0.12	0.03	0.63	-0.003	0.96
rs7105934	-	69239741	A/G	-0.15	0.73	-0.08	0.51	-0.28	0.05
rs718314	*ITPR2*	26453283	A/G	0.31	0.13	0.04	0.53	-0.11	0.11
rs1049380	*ITPR2*	26489544	A/C	0.35	0.07	0.09	0.09	-0.02	0.71
rs10771279	*ITPR2*	26530543	T/C	0.34	0.08	0.11	0.046	-0.02	0.78
rs4765623	*SCARB1*	125320850	T/C	-0.25	0.17	-0.09	0.09	0.05	0.42

a: The association tests between SNPs and tumor size, SNPs and T staging, SNPs and Fuhrman grade were performed by using linear regression.

Supplementary Table 3. Quality control among different genotyping platforms and imputation.

Groups	No.	MassARRAY iPLEX		TaqMan MGB		Concordance rate (imputation)
		Call rate	Concordance rate (Illumina Human OmniExpress Bead Chips)	Call rate	Concordance rate rate (Illumina Human OmniExpress Bead Chips)	
Control	360	98.88%	100%	98.88%	100%	99.79%
